# Planetary health education in practice: public health, climate change, and transdisciplinary learning at University of New England

**DOI:** 10.3389/fpubh.2025.1736662

**Published:** 2026-01-14

**Authors:** Alethea Cariddi, Collyn Baeder, Michelle Cote, Kin Ly, Kris Hall

**Affiliations:** 1 Office of Sustainability, University of New England, Biddeford, ME, United States; 2 Student Wellness and Health Resources, Colby College, Waterville, ME, United States; 3 Center to Advance Interprofessional Education and Practice, University of New England, Portland, ME, United States; 4 Department of Biomedical Sciences, College of Osteopathic Medicine, University of New England, Portland, ME, United States

**Keywords:** environmental health, interprofessional education, planetary health, sustainability, transdisciplinary learning, public health, health professions, climate change

## Abstract

The accelerating impacts of climate change, pollution, and biodiversity loss demand an educational paradigm that integrates ecological and human health systems. The University of New England has implemented a transdisciplinary Planetary Health framework to connect public health, environmental sciences, and health professions. Between 2020 and 2025, UNE’s Planetary Health Council and the Center to Advance Interprofessional Education and Practice co-hosted a series of online and in-person events addressing interconnected issues such as pandemic resilience, environmental injustice, chemical pollution, and biodiversity loss, among others. Post-event surveys from 502 active participants in five events demonstrated strong engagement and positive perceptions of the event format. Respondents frequently cited appreciation for diverse disciplinary perspectives, relevance to professional practice, and delivery format. Suggestions for improvement focused on expanding discussion time and providing deeper topic exploration. Participation data further indicated that virtual and hybrid delivery formats enhanced accessibility and broadened engagement across disciplines. This descriptive retrospective study offers practical insights for educators designing planetary health and interprofessional programming. By documenting participant experience and engagement patterns, this article contributes to the emerging practice-based literature on scalable, transdisciplinary approaches to planetary health education and suggests directions for future research.

## 1 Introduction

The mounting convergence of environmental degradation, climate change, and global health crises underscores the urgent need for educational models that prepare future professionals to think and act across disciplines (1). The emerging field of Planetary Health (2), which recognizes the interdependence between human health and the state of natural systems, calls for transformative approaches to health education that bridge ecological, social and biomedical domains. More attention was brought to the field of Interprofessional Education and Practice in 2010 when the World Health Organization (3) published guidelines for “innovative, system transforming solutions…” championing interprofessional approaches to address global health workforce inequities. Academic institutions are increasingly tasked with equipping learners to address complex challenges such as pollution, food insecurity, biodiversity loss, and climate-driven disease through systems thinking and interprofessional collaboration (4).

The University of New England (UNE), a health sciences university, rooted in the marine and environmental sciences and humanities, responded to this call through the implementation of a transdisciplinary Planetary Health event series. UNE’s Planetary Health Council (PHC) provides an institutional platform to integrate environmental sustainability and public health into curriculum, campus culture, and community partnerships. In collaboration with the Center to Advance Interprofessional Education and Practice (CAIEP), the PHC developed a multi-year series of educational events, designed to strengthen the links between ecological and human health. These events were integrated into CAIEP’s Knowledge Exchange series to promote interprofessional dialogue across disciplines and to expose interprofessional learners to a variety of contemporary issues in planetary health. CAIEP’s Knowledge Exchange events focus on social and environmental drivers of health, which are critical for health professions learners to understand in order to perform at the top of their scope in a clinical team setting (5, 6).

Between 2020 and 2025, five signature events engaged students, faculty, and community members in examining the interconnected systems driving public and planetary health outcomes (7). Each event emphasized multiple perspectives from public health, healthcare disciplines, social work, environmental studies, law, and community advocacy around a specific topic.

The goal of the event series was to introduce learners to key planetary health topics, highlight the relevance of environmental degradation to health professions and environmental students, and foster dialogue across fields around complex public health challenges. The purpose of this article is to describe the design and implementation of this event-based educational model and examine participant feedback to understand perceived engagement, topic relevance, and the strength and limitations of different delivery formats using thematic analysis of qualitative data. By documenting participant perspectives across multiple events, this article situates UNE’s approach within the growing call for planetary health content in interprofessional and interdisciplinary education (8) and offers practice-based insights for educators seeking accessible ways to introduce planetary health concepts into co-curricular opportunities.

## 2 Pedagogical frameworks, principles, and competencies

Although the four frameworks described below emerge from different domains, they share several core commitments including integrating diverse perspectives, fostering systems-thinking, and preparing learners to address complex, real-world challenges. The event series was developed pragmatically to meet institutional goals and student interest, rather than designed around a single pedagogical framework. Because of the descriptive retrospective study design, no one framework fully captures the breadth of the learning objectives addressed across the events. Instead, these complementary frameworks collectively illuminate the educational intent and contextualize the series to demonstrate its alignment with widely recognized competencies (9) in planetary health, public health, and interprofessional education (IPE).

### 2.1 Transdisciplinary learning framework

The event series applied transdisciplinary pedagogy by intentionally convening experts and learners from diverse fields, such as public health, health care, environmental science, sociology, policy, and others, to co-create knowledge. This approach aligns with pedagogical models that promote integration across disciplinary boundaries to foster innovative, systems-level solutions to complex issues like climate change and human and environmental health (9).

### 2.2 Interprofessional education principles

Drawing on the Interprofessional Education Collaborative (IPEC) Core Competencies (10), of Values and Ethics, Roles and Responsibilities, Communication, and Teams and Teamwork, the events provided experiential learning opportunities for health professions students and faculty to engage with peers in health and non-health disciplines. The collaboration with CAIEP ensured alignment with national IPE standards.

### 2.3 Planetary health education framework

The structure and content of the series reflected key competencies identified by the Planetary Health Alliance and the Planetary Health Competency Framework: interconnection within Nature, the Anthropocene and health, equity and social justice, movement building and systems change, and systems thinking and complexity (11, 12). These competencies informed the thematic design of each event.

### 2.4 Alignment with public health education standards

The educational design of these events also aligns with the Core Competencies for Public Health Professionals developed by the Council on Linkages Between Academia and Public Health Practice (13). These competencies emphasize interdisciplinary collaboration, systems thinking, and the integration of evidence-based decision-making into public health practice. The UNE Planetary Health series reflected several domains of this framework by creating opportunities for participants to engage with complex, real-world health and environmental challenges through transdisciplinary dialogue.

## 3 Learning environment(s)

UNE’s Planetary Health event series was delivered across multiple formats to maximize accessibility and interdisciplinary participation. The first two events, *Pandemic Reflections: Protecting Public Health from COVID to Climate Change* and *Cracking the Zip Code: Decoding Environmental Injustice*, were both held virtually via Zoom, Facebook Live, and UNE livestream, with recordings made publicly available on YouTube to extend reach beyond immediate participants. The third event, *Exposing Forever Chemicals: PFAS Contamination in Maine (Part 1)*, transitioned to an in-person-only setting, allowing direct engagement among attendees, while also being recorded and shared online for asynchronous learning. The fourth event, *PFAS Part 2*, adopted a hybrid model, combining in-person participation with simultaneous Zoom and UNE livestream access, enabling both campus and remote audiences to interact with panelists in real time. It was also recorded and available for viewing later. The final event, *Buzzkill: The Impact of Pollinator Decline on Food Diversity and Human Health*, returned to a fully online format through Zoom, maintaining inclusivity across UNE’s geographically distributed campuses and beyond through a recorded YouTube version.

## 4 Educational design

Each PHC-CAIEP event followed a consistent developmental process. The PHC team began by identifying timely topics at the intersection of environment and health, drawing on faculty expertise, current research, and community health priorities. Once a topic was selected, the team identified and invited three speakers whose areas of expertise represented distinct disciplines or professional perspectives. For example, in the first PFAS-related event, *“Exposing Forever Chemicals: PFAS Contamination in Maine, Part 1,”* our speakers included a chemist, a physician, and the advocacy director from Defend Our Health, a nonprofit, environmental and public health advocacy organization. They were able to provide background expertise about PFAS chemicals and their environmental persistence, explore the consequential human health implications of these chemicals, and describe the legislative advocacy and decision-making surrounding PFAS, respectively. Table 1 summarizes the expertise of each speaker and the perspective they represented for each event, demonstrating the range of perspective diversity.

**Table 1:** Panelist expertise and perspective representation by event.

Events	Expertise/Perspective	Speaker 1	Speaker 2	Speaker 3
*Pandemic Reflections: Protecting Public Health, from COVID to Climate Change*	Expertise:	Microbiologist and infectious disease	Epidemiologist and public health	Public policy and sustainable development leadership
	Representative Perspective:	Tracing the COVID-19 pandemic and epidemiological origins	Parallels between pandemic and climate crises and the role of public health in both	Risk and inequity of pandemics and climate change and adaptive resilience with sustainable development
*Cracking the Zip Code: Decoding Environmental Injustice*	Expertise:	Public health nursing	Sociologist with expertise on race in science and medicine	Environmental scientist with expertise on environmental racism
	Representative Perspective:	Social determinants of health and historical racist policy decisions impacting health disparities	Racist narratives in science with case study of Flint, MI water crisis	History of environmental justice movement and advocacy
*Exposing Forever Chemicals: PFAS Contamination in Maine, Part 1*	Expertise:	Chemist with expertise in environmental health and safety	Doctor of osteopathic medicine and internist	Advocacy director for policy non-profit organization
	Representative perspective:	Chemical persistence of PFAS	Human health impacts of PFAS	Maine policy updates regarding PFAS
*Exposing Forever Chemicals: PFAS Contamination in Maine, Part 2*	Expertise:	Non-profit organization manager providing direct farmer support	Director for non-profit organization protecting farmland in Maine	State senator
	Representative Perspective:	Agricultural impacts of PFAS	Economic support for farmers and research	Legislation in Maine to address PFAS
*Buzzkill: The Impact of Pollinator Decline on Food Diversity and Human Health*	Expertise:	Ecologist researching pollinators	Registered dietician	Small business owner using local honey in products
	Representative perspective:	Environmental health	Human nutrition and health	Economic viability of sustainable business

PHC members then worked closely with the invited speakers to shape the presentations, ensuring that each contribution fit within the event’s structure and collectively formed a coherent narrative. To support continuity and clarity, the PHC team prepared integrated slide decks and coordinated speaker transitions so the presentation would flow smoothly from one perspective to another. After the three speakers, time was intentionally safeguarded for open-ended audience Q&A, as well as a few minutes for closing remarks.

CAIEP staff supported the production and implementation of each PHC event through a range of logistical and technical contributions. This included organizing rehearsals to refining timing and presentation flow, promoting events across campus channels, and coordinating with UNE’s Communications team to manage audiovisual production, livestreaming and video recording. CAIEP staff and PHC members collaborated on identification of the IPE Core Competencies highlighted in each event, which are summarized in Table 2. CAIEP also developed the post-event attendance and feedback surveys used to evaluate audience engagement, perceived learning, and the effectiveness of interprofessional formats. De-identified survey data were later provided by CAIEP for analysis and inclusion in this article.

**Table 2:** IPEC core competencies identified by program coordinators per event.

Events	IPEC core competencies			
	Values and ethics	Roles and responsibilities	Communication	Teams and teamwork
*Pandemic Reflections: Protecting Public Health, from COVID to Climate Change*	X			X
*Cracking the Zip Code: Decoding Environmental Injustice*	X		X	
*Exposing Forever Chemicals: PFAS Contamination in Maine, Part 1*	X	X		
*Exposing Forever Chemicals: PFAS Contamination in Maine, Part 2*	X	X		
*Buzzkill: The Impact of Pollinator Decline on Food Diversity and Human Health*	X	X		

## 5 Evaluation methods

In addition to the active participants captured in our survey sample, the event series reached a much broader audience through passive viewing on Facebook Live, UNE livestream, and YouTube. These broadcast formats substantially expanded the visibility of each event and likely introduced planetary health topics to individuals who may not have otherwise engaged. However, metrics from passive-viewing platforms only capture exposure rather than verified participation, and viewing duration cannot be confirmed. Consequently, survey responses from these platforms cannot be confidently interpreted as evidence of perceived value and were, therefore, excluded from the data analysis. The findings presented here reflect only participants who attended through interactive formats: Zoom and in-person attendance.

### 5.1 Qualitative analysis of open-ended survey responses

Participant feedback was collected through post-event surveys developed and administered by CAIEP. At the conclusion of each event, participants were invited to complete the survey via a QR code displayed on the final slide and through a survey link shared in the Zoom chat; these brought participants to the survey in Google Forms, where the surveys were completed voluntarily.

The survey included both quantitative and qualitative items. The quantitative questions asked respondents to identify their primary academic major or professional discipline. Demographic data was not collected about the participants. The qualitative component consisted of two open-ended prompts: “*Please let us know what was successful about today’s event”* and *“Please let us know what you would change about today’s event.”*

Survey data were provided by CAIEP for analysis in an Excel worksheet with timestamps of each survey submission. The data was de-identified, protecting confidentiality. All data is preserved in a UNE Google Drive with access restricted to key CAIEP staff only. UNE’s Office of Research Integrity determined that this project did not require Institutional Review Board review and approval because it does not involve research with human subjects as defined by 45 CFR 46.102.

De-identified, open-ended responses from post-event surveys were analyzed using an inductive thematic analysis approach guided by established qualitative research methodologies (14–16). The primary author (AC) first reviewed all responses to the prompts across all events to gain familiarity with the data and identify recurring patterns and concepts. This process resulted in a preliminary set of thematic categories reflecting common dimensions of participant feedback.

### 5.2 Coding strategy and treatment of multi-thematic responses

To refine and validate the preliminary coding framework, the primary author conducted a pilot coding exercise using responses from a single event (*PFAS, Part 2*). These responses were manually coded relative to the initial theme categories, allowing for clarification of theme boundaries and ensuring that the categories were simultaneously distinct and inclusive.

After this manual process, the same dataset was analyzed using a large language model (LLM), ChatGPT-5, to independently identify emergent themes. The themes generated by the model were compared with the primary author’s preliminary categories and were found to be conceptually consistent, supporting the clarity and coherence of the initial thematic framework.

Following this validation step, open-ended responses from each event were entered sequentially into the LLM, and the model was prompted to identify the themes represented within each event’s response set. The primary author then compared the themes generated across events and determined that the same core categories were consistently present in all datasets. Based on this cross-event comparison, final theme definitions were refined and confirmed by the primary author.

Each individual response was then coded manually by the primary author using the finalized theme categories. Responses were color-coded by event and by theme to support visual inspection. Responses were not constrained to a single category: short or focused comments (e.g., *“Powerpoints were smooth”*) were assigned one theme, when appropriate, while more complex responses (e.g., *“*Var*iety of speakers, timely, like the open discussion opportunity”*) were coded across multiple themes when they reflected several distinct ideas (e.g., comments referencing speaker diversity, topic relevance, and event format). All responses were categorized within the established thematic framework. No comments were excluded due to lack of thematic fit; rather, variability in responses was captured through single- or multi-theme coding.

Following manual coding, the primary author conducted a descriptive tally of the frequency with which each theme appeared within and across event datasets. These counts reflected the number of responses referencing a given theme, recognizing that individual responses could contribute to multiple thematic categories. Frequency summaries were used to support comparative interpretation across events and to contextualize patterns in participant feedback. All frequency determinations were derived from the primary author’s manual coding decisions and analytic judgment.

### 5.3 Use of artificial intelligence as an analytic support tool

This analytic process aligns with widely accepted guidelines for thematic analysis. Consistent with Braun and Clarke’s six-phase approach (14), the primary author engaged in repeated familiarization with the data, generated initial codes through iterative review and pilot coding, developed and reviewed themes across event datasets with AI-assisted pattern checking, defined and refined final thematic categories through cross-event comparison, and produced the final analytic narrative through manual coding and synthesis of representative responses.

Artificial intelligence was used as a supportive analytic tool to assist with pattern recognition and theme confirmation. The LLM was employed after initial theme development to corroborate emergent categories, assess consistency of themes across event datasets, and enhance analytic efficiency, given the volume of responses. All thematic decisions, final category definitions, and coding assignments were determined by the primary author through critical review.

This approach is consistent with emerging literature that supports the responsible use of generative AI in qualitative research when applied transparently and under human oversight (17, 18). Prior studies have demonstrated that AI-assisted thematic analysis can enhance rigor and scalability while maintaining analytic credibility when researchers retain control over interpretation, reflexivity, and final decision-making (19, 20). In this study, AI functioned as an analytic aid rather than an interpretive authority, ensuring that thematic conclusions remained grounded in researcher judgment and contextual understanding.

## 6 Results

### 6.1 Overview of participant engagement

Across the five PHC events, a total of 767 participants engaged meaningfully in active formats, including Zoom and in-person attendance. Learners included primarily undergraduate and graduate students from the health professions, environmental and social sciences, as well as faculty, professional staff, and occasional community participants. Collectively, 502 post-event surveys were gathered (a 65.4% response rate), representing participants from over 23 distinct academic programs and professional disciplines across UNE. One survey from the first event, four surveys from the second event, and one survey from the fourth event were excluded from the dataset because their timestamps indicated completion in the days and months following the live session, revealing they were submitted by passive viewers, rather than by active participants. Active participation, as well as passive viewing, program/discipline representation, and number of surveys collected are summarized in Table 3. Attendance varied by delivery format, with the highest participation observed in fully virtual events, and the lowest in the sole in-person-only session.

**Table 3:** Total event attendance, participation, and survey completion by academic program.

Survey responses by participant program	Pandemic reflections: protecting public health, from COVID to climate change (10/15/2020)	Cracking the zip code: decoding environmental injustice (9/15/2021)	Exposing forever chemicals: PFAS contamination in Maine, Part 1 (4/12/2023)	Exposing forever chemicals: PFAS contamination in Maine, Part 2 (9/20/2023)	Buzzkill: the impact of pollinator decline on food diversity and human health (4/2/2025)
Number of active participants	128	230	35	177	197
Total surveys from active participants	49	132	16	125	180
**Surveys submitted by program/discipline**					
Animal behavior	1				
Dental hygiene	2	1	1		10
Dental medicine	1				
Environmental science			10	6	11
Environmental studies		3	1		
Health informatics		1			
Health, wellness and occupational studies	1	8			
Marine affairs	2	1		1	
Marine biology	3				
Marine science		2	2	8	1
Medical biology		3	1	4	1
Neuroscience	1				
Occupational therapy	1	8			
Osteopathic medicine	24	79		91	144
Pharmacy	3	14			
Physical therapy		2		4	
Physician assistant	1	1			
Psychology	2	1	1		
Public health	2			1	
Nursing	2	3		2	
Nutrition		1		2	3
Social work	1	2			1
Sustainability and business				1	
Other	2	2		5	9
Passive viewers (as of December 17, 2025)	246	498	12,451	1,437	109

### 6.2 Participant perceptions

Of the 502 post-event surveys collected across the five PHC events, 312 (62.2% of survey respondents) participants provided open-ended responses to the prompt *“Please let us know what was successful about today’s event*,*”* and 235 (46.8% of survey respondents) participants responded to *“Please let us know what you would change about today’s event*.*”* These qualitative data formed the basis of the thematic investigation.

#### 6.2.1 Participant perception of event success

Analysis of the 312 open-ended responses to the prompt *“Please let us know what was successful about today’s event”* revealed five overarching themes: (1) Engaging and knowledgeable panelists, (2) Effective event design and flow, (3) Compelling and informative content, (4) Value of multiple perspectives, and (5) Relevance to practice and everyday life. Table 4 summarizes participant perception of event successes through each of these themes.

**Table 4:** Participant perception of event success.

Participants, survey completion and responses	Pandemic reflections: protecting public health, from COVID to climate change	Cracking the zip code: decoding environmental injustice	Exposing forever chemicals: PFAS contamination in Maine, Part 1	Exposing forever chemicals: PFAS contamination in Maine, Part 2	Buzzkill: the impact of pollinator decline on food diversity and human health
Number of active participants	128	230	35	177	197
Completed surveys from active participants	49	132	16	125	180
Surveys with success responses	37	96	10	80	89

Feedback themes	Frequency of responses coded by theme				
Engaging and knowledgeable panelists	11	21	5	21	27
Effective event design and flow	12	32	3	23	10
Compelling and informative content	20	67	4	38	49
Value of multiple perspectives	10	19	2	16	22
Relevance to practice and everyday life	4	15	0	26	15

Participants frequently praised the engaging and knowledgeable panelists, highlighting their expertise, clarity, and enthusiasm. Respondents noted that the speakers’ diverse experiences and ability to translate complex information fostered connection and credibility (e.g., *“All three presenters had great information to share and were very engaging. The passion and dedication of these individuals is evident*.*”* and *“The speakers were all very knowledgeable about the topic and had a lot of insightful information to share.”*).

The second theme, effective event design and flow, reflected appreciation for the organization and pacing of the session. Participants commented on the smooth transitions between speakers, clear moderation, and opportunities for discussion or questions (e.g., *“I enjoyed the q&a after the speakers wrapped up.”* and *“Clear flow of the panelists in their respective parts.”*). Some also expressed appreciation for resource links shared in the chat function of Zoom (e.g., *“I appreciated all of the active, real-time sharing of links in the chat.”*).

Feedback related to event content highlighted participants’ appreciation for the interest, novelty, and depth of the topics explored. Many attendees described the sessions as *“very informative,” “fascinating,”* and *“an enlightening experience.”* Rather than focusing solely on knowledge gain, participants’ comments conveyed genuine interest and enjoyment, suggesting that the delivery format and topics chosen effectively captured attention and inspired reflection (e.g., *“I really enjoyed the comparison between covid and climate change specifically as it related to inequalities.”* and *“They explained a complicated topic in simple effective ways.”*).

The fourth theme, value of multiple perspectives, captured participants’ recognition of the interdisciplinary format. Respondents expressed appreciation for hearing from speakers across varied fields (e.g., “*The use of a variety of focuses and viewpoints/expertise to examine the topic holistically from any angles help me feel like I was able to make stronger connections and develop a deeper understanding of the material.”* and *“A broad range of views and experiences as well as backgrounds that allowed an interdisciplinary discussion.”*).

Finally, the theme of relevance to practice and everyday life reflected comments connecting event topics to professional roles, personal behaviors, or local contexts. Participants described the sessions as *“eye-opening”* and *“applicable to future healthcare practice,”* indicating that they perceived clear links between planetary health concepts and real-world application (e.g., *“Loved the emphasis on providing healthy food to our patients.”* and *“I thought the use of empirical data and real-life examples was very powerful in showing how the region you live in can affect your overall health.”*).

#### 6.2.2 Participant suggestions for improvements

Analysis of the 235 open-ended responses to the prompt *“Please let us know what you would change about today’s event”* revealed five overarching themes of constructive feedback: (1) Delivery format, (2) Presentation style, (3) Desire for greater depth, (4) Opportunities for engagement, and (5) Satisfaction with event. Table 5 summarizes participant suggestions for improvements through each of these themes.

**Table 5:** Participant suggestions for improvements.

Participants, survey completion and responses	Pandemic reflections: protecting public health, from COVID to climate change	Cracking the zip code: decoding environmental injustice	Exposing forever chemicals: PFAS contamination in Maine, Part 1	Exposing forever chemicals: PFAS contamination in Maine, Part 2	Buzzkill: the impact of pollinator decline on food diversity and human health
Number of active participants	128	230	35	177	197
Completed surveys of active participants	49	132	16	125	180
Surveys with improvement responses	31	79	9	55	61

Feedback themes	Frequency of responses coded by theme				
Delivery format	2	12	2	8	3
Presentation style	1	10	0	0	2
Desire for greater depth	4	7	2	13	9
Opportunities for engagement	5	13	1	11	4
Satisfaction with event	20	37	4	24	61

Comments about delivery format reflected logistical preferences, such as desires for holding the event at a different *“time of day,”* or that they *“wish we could be in person.”* For example, one participant wrote, *“I would push the event back by 15 min if possible,”* while another noted, *“I think hosting the event in a larger room on campus would allow for more people to participate in person.”*

Feedback related to presentation style included recommendations to *“provide the slides in advance”* or comments referring to time constraints, like *“felt rushed.”* Included in this thematic category were comments like, *“I would love an acknowledgement of the indigenous land we are currently on.”* and *“I know that time is very limited, but if speakers could talk a little slower that would be great.”*

Several participants expressed a desire for more depth, noting that they “*wanted more specific data,”* or cited specific information pertinent to the topic that they wanted to learn more about, like one participant who said, *“I would have loved to hear testimonials directly from the farmers about how this work is affecting them. I would also have loved if they gave more specific details/graphics of PFAS contamination in Maine.”*

Others emphasized the value of interactive engagement, proposing more audience Q&A or small-group discussions to enhance connection and dialogue, e.g., *“More time for discussion and questions.”* and *“Opportunity to talk to other students on the chat (Breakout sessions).”*

Finally, a substantial portion of respondents (62%) indicated no desired changes, commenting simply, *“nothing,” “it was great,”* or *“no improvements needed,”* underscoring overall satisfaction with the event experience.

## 7 Discussion

### 7.1 Overview of participant engagement

Across the five PHC events, survey completion patterns evolved over time. Increased response rates in later events may reflect participants’ growing familiarity with the event format and organizers’ more explicit emphasis on the importance of feedback for strengthening future programming. These improvements suggest that consistent messaging and integration of feedback culture into educational events can meaningfully enhance participation and the quality of evaluative data.

Attendance and participation patterns also offer insights for optimizing future delivery formats. While several respondents from virtual sessions expressed desire for in-person gatherings to encourage interpersonal connections, the attendance data suggest that virtual and hybrid formats were significantly more accessible and inclusive. The sole in-person-only event drew the smallest audience and generated limited survey data, offering insufficient evidence to support a transition to face-to-face programming alone. These findings highlight an important balance between accessibility and experiential connection, suggesting that hybrid approaches, which allow for both physical presence and remote participation, may best support broad engagement in planetary health education.

### 7.2 Participant perceptions

#### 7.2.1 Participant perceptions of event success

Analysis of post-event survey data revealed that participants consistently valued opportunities to engage with diverse perspectives on planetary health topics. The emphasis on multiple viewpoints, collaborative dialogue, and real-world application reflected the success of the events’ design in fostering systems-based thinking and transdisciplinary awareness. Learners expressed appreciation for the relevance of topics and the cohesion of the panel format, affirming that the intentional pairing of experts from different fields supported meaningful synthesis across disciplines. The emphasis on enjoyment, engagement, and discovery indicates that these events effectively fostered intellectual curiosity and motivation.

#### 7.2.2 Participant suggestions for improvements

Participant feedback also points to actionable ways to enhance future planetary health programming. Constructive feedback emphasized participants’ desire for even more discussion time, deeper exploration of content, and increased opportunities for interaction. These preferences stress the importance of active learning strategies within public and planetary health education, where dialogue and reflection reinforce knowledge integration. These suggestions did not reflect dissatisfaction with the event themselves, but rather enthusiasm for extending and deepening the experience. Future iterations could integrate breakout discussions, case-based applications, or follow-up workshops to sustain engagement and deepen understanding beyond the event itself.

### 7.3 Limitations

Interpretation of these findings should be considered in light of several important limitations. The voluntary nature of participation in the PHC event series introduces potential participation bias. Individuals who elect to attend may represent a group already interested in planetary health, interdisciplinary learning, or climate-related issues. A further source of participation bias stems from institutional requirements and elective incentives within the medical program. Beginning in 2022, first-year medical students were required to attend at least three CAIEP Knowledge Exchange events per academic year, and second-year students were required to attend at least two per academic year. In addition, many medical students voluntarily pursue the Interprofessional Honors Distinction, a nationally accredited digital credential, which requires attendance at four CAIEP events. These expectations likely contribute to higher participation rates among medical students relative to other disciplines and may partially explain the strong representation of this group in the survey data. While this engagement aligns with curricular goals, it also introduces an attendance pattern influenced by program requirements rather than solely by learner interest.

The descriptive nature of the study design further limits interpretation. Without demographic data, it is impossible to assess the representativeness of the event participants or survey respondents, leading to uncertain generalizability. Survey responses may also reflect response bias, in which participants with particularly favorable feedback (or conversely, particularly critical feedback) are more likely to respond. Additionally, feedback was not collected from passive viewers of the event recordings, resulting in a data gap from this segment of the audience.

Beyond these limitations of data collection, limitations related to analytical rigor warrant consideration. While the primary author reviewed, refined, and consolidated the resultant themes, the reliance on generative AI as an analytic support tool, as well as the lack of a second reviewing author, may limit the rigor of the inductive thematic analysis (21). Despite this, emerging evidence supports the cautious and transparent use of generative AI to assist with qualitative pattern recognition when paired with vital human oversight (22). Therefore, the findings should be interpreted as exploratory and descriptive, rather than definitive.

### 7.4 Implications for future direction

Despite the limitations, these findings offer meaningful insights into the design and delivery of planetary health education in higher education settings. The PHC-CAIEP partnership model demonstrates a scalable framework for convening diverse disciplinary perspectives around complex health-environment challenges in ways that are accessible, engaging, and responsive to learner feedback. The consistent appreciation for interdisciplinary dialogue, coupled with strong participation in virtual formats, suggests that similar models could be adapted across universities seeking to embed planetary health content into co-curricular settings.

Future research should build on this work by incorporating more systematic outcomes measures, including longitudinal assessment of learning, content application, and systems-thinking decision-making, with the addition of demographic data to assess representativeness and differential impacts across learner groups. Mixed-methods designs, multiple coders, and intentional alignment with a single guiding framework from the start may further strengthen analytical rigor. Together, these next steps can advance planetary health education from exploratory programs to evidence-based educational practice.

## 8 Conclusion

This descriptive analysis of UNE’s Planetary Health event series provides insight into how an event-based, transdisciplinary educational model can be experienced by learners across diverse academic programs. Participant feedback consistently highlighted appreciation for a variety of perspectives, clear organization, and real-world connections shared by speakers. Active participation data showed that flexible delivery formats, specifically virtual and hybrid options, increased accessibility and participation.

Importantly, this study did not assess learning outcomes, behavior change, or longer-term impacts, and findings are limited to self-reported perceptions from a voluntary sample. Nonetheless, the results offer practical guidance for educators designing planetary health programming, particularly regarding speaker selection, event structure, and delivery format. Future research should build on this work by examining learning outcomes, longitudinal impacts, and how planetary health content can be more intentionally integrated into formal interprofessional curricula. Together, these findings contribute to an emerging practice-based literature on feasible, scalable approaches to introducing planetary health concepts in higher education.

## Data Availability

The raw data supporting the conclusions of this article will be made available by the authors, without undue reservation.
